# The association between prolonged sedentary time and coronary artery calcification in young healthy men in Korea: a cohort study

**DOI:** 10.1038/s41598-022-06739-x

**Published:** 2022-02-17

**Authors:** Min-Woo Nam, Yesung Lee, Woncheol Lee

**Affiliations:** Department of Occupational and Environmental Medicine, Kangbuk Samsung Hospital, Sungkyunkwan University School of Medicine, 29 Saemunan-ro, Jongno-gu, Seoul, 03181 South Korea

**Keywords:** Cardiology, Health care, Medical research

## Abstract

Coronary artery calcium score (CACS) is a useful method for predicting coronary artery disease in asymptomatic adults. In this study, we investigated the association between prolonged sedentary time and CACS. A cohort study was conducted in 14949 men with negative CACS (CACS = 0) at baseline who were followed up at least once. Sedentary time was categorized into < 7, 7–8, and ≥ 9 h/day. CACS was calculated by cardiac tomography. During 60,112.1 person-years of follow-up, 569 participants developed positive CACS. The multivariable adjusted hazard ratios (95% confidence intervals) for incident positive CACS comparing sedentary times of 7–8 h/day and ≥ 9 h/day to sedentary time of < 7 h/day were 1.25 (0.97–1.62) and 1.28 (1.03–1.59), respectively. This association was more strongly observed in the non-obese group (BMI < 25 kg/m^2^). In contrast, in the obese group (BMI ≥ 25 kg/m^2^), there was no significant association between sedentary time and incidence of positive CACS. Prolonged sedentary time was significantly associated with incidence of positive CACS in the study. CACS is also an effective screening tool for predicting future cardiovascular events in asymptomatic patients. Therefore, CACS can be an effective screening method for predicting coronary artery diseases in people with prolonged sedentary time, especially in metabolically healthy people.

## Introduction

Nowadays, people spend prolonged periods sitting down while using electronic communication equipment and computers. According to the 2016–2018 Korea National Health & Nutrition Examination Survey^1^, men and women spend an average of 8.2 h and 8.1 h per day, respectively, sitting down. Prolonged sedentary time has a significant correlation with type 2 diabetes, the prevalence of cardiovascular disease (CVD), cancer, and even all-cause mortality^2^. In addition, sedentary workers often complain of musculoskeletal disorders^3,4^ that are accompanied by pain and disability.

According to the 2019 cause of death statistics in Korea^5^, heart disease ranks second after cancer among the causes of death in Koreans, accounting for 10.5% of the total. The coronary artery calcium score (CACS) is a widely used method for evaluating coronary artery disease and has been thoroughly studied^6^. According to a 6-year prospective multicenter study^7^, there was no significant difference in the diagnosis and prediction of future cardiovascular events in CACS evaluation compared to coronary computed tomographic angiography in asymptomatic adults. Thus, CACS is an effective tool for predicting cardiovascular events in asymptomatic adults. In this study, we investigated the association between sedentary time and CACS and analyzed the effect of prolonged sedentary time on coronary artery disease. We also conducted a large-scale study in a relatively healthy adult male group using a cohort established from regular health screening data.

## Methods

### Study population

The Kangbuk Samsung Health Study^8^ is a cohort study of men and women who underwent a comprehensive health examination at the Kangbuk Samsung Hospital Total Healthcare Center. This study analyzes the health examination results of healthy people for prolonged periods to understand the health level of Koreans and to create basic data for predicting and preventing future health problems. This was a retrospective cohort study from January 2002 to March 2012, and the Kangbuk Samsung Hospital officially started a prospective cohort study in April 2012.

**Figure 1:**
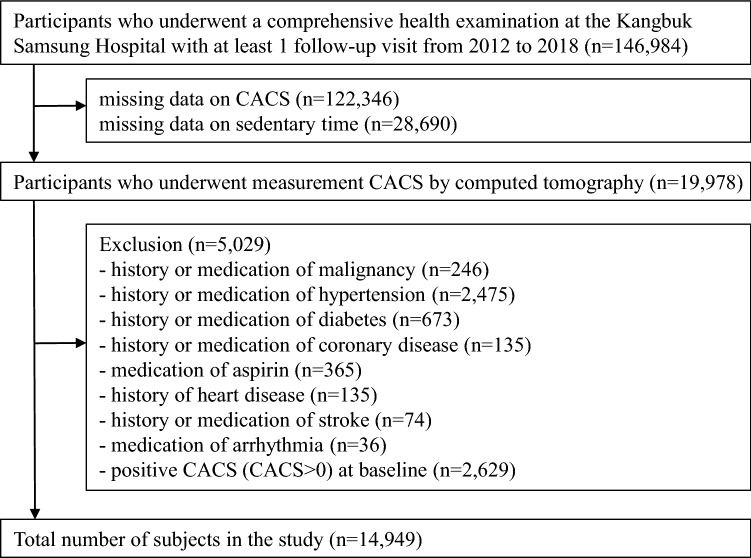
Flow chart of study participants.

The present study included all male participants who underwent a comprehensive health examination at the Kangbuk Samsung Hospital with at least one follow-up visit from 2012 to 2018 (n = 146984). We initially selected men and women for the analysis; however, in the case of women, the number of incident cases was too small, thus excluding them from the final analysis. And, we excluded participants without data on CACS or sedentary time: missing data on CACS (n = 122346); missing data on sedentary time (n = 28690). In addition, we excluded participants who had any of the following conditions at baseline: history or medication of malignancy (n = 246); history or medication of hypertension (n = 2475); history or medication of diabetes (n = 673); history or medication of coronary disease (n = 135); medication with aspirin (n = 365); history of heart disease (n = 135); history or medication of stroke (n = 74); medication of arrhythmia (n = 36); and positive CACS (CACS > 0) at baseline (n = 2629). Finally, the total number of participants in the study was 14949 (Fig. 1).

We followed the practice of the Declaration of Helsinki and this study was approved by the Institutional Review Board (IRB) of Kangbuk Samsung Hospital (KBSMC 2021-06-019), which waived the requirement for written informed consent due to the use of anonymized data obtained as part of regular medical examinations.

### Data collection

All examinations were conducted at the Kangbuk Samsung Hospital Total Healthcare Screening Center clinics in Seoul and Suwon, South Korea. Participants underwent a comprehensive health examination every year or biennial. The comprehensive health examination includes basic tests such as blood tests and urine tests, and optional test such as computer tomography (CT), Magnetic Resonance Imaging (MRI), and endoscopy. At each clinical visit, the medical history, medication, sedentary time, alcohol consumption, smoking status, and exercise were collected using standardized questionnaires. The total weekday sedentary time was measured by the single question, “During the last 7 days, how much time did you usually spend sitting on a weekday?” This estimate included the time spent sitting in a variety of domains (i.e., work and leisure). Currently, there are no well-accepted thresholds for data presented as categorical levels^9^. Then, considering that the average daily working time of Korean office worker is 8 h, 7–8 h/day were organized into one group, and all participants were divided into 3 groups, including those above (≥ 9 h/day) and below (< 7 h/day). According to the Korea Labor Standards Act, working time of adults should not exceed 40 h per week excluding recess hours (12 additional hours per week are allowed with workers’ permission)^10^.

Alcohol consumption was categorized into nondrinking, light drinking (< 10 g/day), moderate drinking (10–30 g/day), and heavy drinking (≥ 30 g/day). Smoking status was categorized as never, former, or current smoker. We asked participants in the questionnaires, “During the last 7 days, on how many days did you do vigorous physical activities like running, aerobics, riding a bicycle fast?” We defined that participants who answered that they had vigorous physical activity for more than 3 days per week did “regular exercise.” Height and weight were measured by well-trained nurses, with the participants wearing a lightweight hospital gown and no shoes. Height was measured to the nearest 1 mm using a stadiometer with the participant standing barefoot. Weight was measured to the nearest 0.1 kg on a bioimpedance analyzer (InBody 3.0, Inbody 720, Biospace Co., Seoul, Korea). Body mass index (BMI) was calculated as weight in kilograms divided by height in meters squared. BMI was categorized based on the criteria^11^ established for Asian populations: underweight, BMI < 18.5 kg/m^2^; normal-weight, BMI = 18.5–22.9 kg/m^2^; overweight, BMI = 23–24.9 kg/m^2^; obesity, BMI ≥ 25 kg/m^2^. Blood pressure (BP) was measured by trained nurses while participants were in a sitting position with the arm supported at the heart level.

### Laboratory analyses

Blood specimens were sampled from the antecubital vein after at least 10 h of fasting. Serum glucose was measured using the hexokinase method on a Cobas Integra 800 apparatus (Roche Diagnostics, Tokyo, Japan). Serum total cholesterol and triglyceride levels were determined using an enzymatic colorimetric assay. Low-density (LDL-C) and high-density lipoprotein cholesterol (HDL-C) levels were measured directly using a homogeneous enzymatic colorimetric assay. Serum high-sensitivity C-reactive protein (hsCRP) levels were determined using a particle-enhanced immunoturbidimetric assay on a Modular Analytics P800 apparatus (Roche Diagnostics, Tokyo, Japan). The Laboratory Medicine Department of the Kangbuk Samsung Hospital is accredited by the Korean Society of Laboratory Medicine (KSLM) and the Korean Association of Quality Assurance for Clinical Laboratories (KAQACL) and is a participant in the College of American Pathologists (CAP) Survey Proficiency Testing.

### Measurement of CACS by multidetector CT

CT scans were performed with a LightSpeed VCT XTe-64-slice multidetector CT scanner (GE Healthcare, Tokyo, Japan) in both Seoul and Suwon centers using the same standard scanning protocol: 2.5-mm thickness, 400-ms rotation time, 120-kV tube voltage, and 124-mA s (310 mA × 0.4 s) tube current under electrocardiogram-gated dose modulation. CACS were calculated as previously described by Agatston et al.^12^. CACS were categorized as either negative CACS (CACS = 0) or positive CACS (CACS > 0) in our study^13,14^.

### Statistical analysis

The characteristics of the study participants were explored according to sedentary time. To test for linear trends, category numbers were used as continuous variables in the regression models. We started follow-up with all participants free of CACS at baseline, and the endpoint was the incident positive of CACS. Follow-up for each participant was extended from the baseline examination until the development of positive CACS or the last health examination conducted prior to December 31, 2018, whichever came first. Incidence rates were calculated as the number of incident cases divided by person-years of follow-up.

The primary analysis was based on categorized sedentary time (< 7, 7–8, and ≥ 9 h/day), and we estimated adjusted hazard ratios (HRs) with 95% confidence intervals (CIs) for incidence of positive CACS, comparing sedentary time categories at baseline with the sedentary time of < 7 h/day. To determine the linear trends of risk, we used a continuous variable with the category number and tested its statistical significance in the regression models. The models were initially adjusted for age and then further adjusted for alcohol intake, smoking status, regular exercise, BMI, systolic BP, glucose, LDL-C, triglycerides, medication for dyslipidemia, and hsCRP. We also conducted a subgroup analysis to identify the interactions between sedentary time and obesity. Statistical significance was set at p < 0.05 (two-tailed). We used STATA version 17.0 (Stata Corp., College Station, TX, USA) for data analysis.

## Results

The baseline characteristics of the study participants are shown in Table 1. The mean (standard deviation) age and BMI of the 14,949 participants were 37.3 (6.0) years and 24.6 (3.0) kg/m^2^, respectively. Participants who reported a sedentary time of ≥ 9 h/day were more likely to be younger, were less likely to exercise and drink alcohol, and had lower levels of systolic and diastolic BP and fasting glucose than those who reported a sedentary time of < 7 h/day.

**Table 1:** Baseline characteristics of study participants by reported sedentary time.

Characteristics	Overall	Sedentary time			*p* for trend
		< 7 h/day	7–8 h/day	≥ 9 h/day	
Number	14949	3646	3205	8098	
Age (years)^a^	37.3 (6.0)	38.9 (6.7)	37.7 (6.0)	36.4 (5.5)	< 0.001
Current smoker (%)	28.38	30.94	27.08	27.75	0.001
Alcohol intake (%)^b^	53.70	58.80	54.29	51.16	< 0.001
Regular exercise (%)^c^	12.66	15.25	13.67	11.09	< 0.001
Obesity (%)	41.05	41.47	40.56	41.05	0.766
BMI (kg/m^2^)	24.6 (3.0)	24.7 (2.9)	24.6 (2.9)	24.6 (3.0)	0.961
Systolic BP (mmHg)^a^	112.5 (10.5)	113.2 (10.7)	112.6 (10.5)	112.1 (10.3)	< 0.001
Diastolic BP (mmHg)^a^	73.0 (8.8)	73.8 (8.9)	73.1 (8.9)	72.5 (8.6)	< 0.001
Fasting glucose (mg/dL)^d^	94.0 (90–99)	95.0 (91–101)	95.0 (90–100)	94.0 (89–99)	< 0.001
Total cholesterol (mg/dL)^a^	200.6 (33.8)	200.7 (34.0)	200.5 (34.5)	200.5 (33.5)	0.805
LDL-C (mg/dL)^a^	130.2 (30.9)	130.2 (31.4)	130.4 (31.2)	130.2 (30.6)	0.866
HDL-C (mg/dL)^a^	52.5 (12.5)	52.7 (12.8)	52.5 (12.4)	52.3 (12.4)	0.075
Triglycerides (mg/dL)^d^	114.0 (82–165)	116.0 (81–167)	114.0 (82–163)	114.0 (82–165)	0.305
Medication for dyslipidemia (%)	1.76	1.84	1.75	1.73	0.692
hsCRP (mg/L)^d^	0.05 (0.03–0.10)	0.05 (0.03–0.11)	0.05 (0.03–0.10)	0.05 (0.03–0.10)	0.356

*BMI* body mass index, *BP* blood pressure, *LDL-C* low-density lipoprotein cholesterol, *HDL-C* high-density lipoprotein cholesterol, *hsCRP* high-sensitivity C-reactive protein.

^a^Data are presented as the means (standard deviation).

^b^Data are presented as percentage (the proportion of participants who intake more than 10 g of alcohol a day).

^c^Data are presented as percentage (the proportion of participants who answered that they had vigorous physical activity for more than 3 days per week).

^d^Data are presented as the median (interquartile range), or percentage.

We identified 569 incidence of positive CACS during 60112.1 person-years of follow-up (incident rate, 9.5 per 1000 person-years). The average follow-up period for participants was 4.0 years. Table 2 shows the association between sedentary time and incidence of positive CACS. In an age-adjusted model, the adjusted HRs (95% CIs) for incidence of positive CACS comparing sedentary time of 7–8 and ≥ 9 h/day to the sedentary time of < 7 h/day were 1.16 (0.91–1.48) and 1.15 (0.94–1.42), respectively. In a multivariate model adjusting for age, alcohol intake, smoking status, regular exercise, BMI, systolic BP, glucose, LDL-C, triglycerides, medication for dyslipidemia, and hsCRP, the adjusted HRs (95% CIs) for incident positive CACS comparing sedentary time 7–8 and ≥ 9 h/day to the sedentary time of < 7 h/day were 1.25 (0.97–1.62) and 1.28 (1.03–1.59), respectively, and there was a significant association between sedentary time and incidence of positive CACS (*p* for trend = 0.036). As a result of performing the same analysis using sedentary time as a continuous variable, HR was 1.025 (1.001–1.050).

**Table 2:** Development of positive coronary artery calcium score (CACS) by sedentary time in study participants.

Sedentary time	Person-years	Number of incident cases	Incidence rate (per 1000 person-years)	Age-adjusted HR	Multivariable-adjusted HR^a^ (95% CI)
< 7 h/day	14260.6	139	9.7	1.00 (reference)	1.00 (reference)
7–8 h/day	12645.7	127	10	1.16 (0.91–1.48)	1.25 (0.97–1.62)
≥ 9 h/day	33205.8	303	9.1	1.15 (0.94–1.42)	1.28 (1.03–1.59)
*p* for trend				0.205	0.036
Sedentary time (as a continuous variable)				1.02 (0.995–1.042)	1.03 (1.001–1.050)

*CI* confidence interval, *HR* hazard ratio.

^a^Adjusted for age, alcohol intake, smoking status, regular exercise, BMI, systolic blood pressure, glucose, LDL, triglycerides, medication for dyslipidemia, and hsCRP level.

This association was more strongly observed in non-obese participants (BMI < 25 kg/m^2^). In non-obese participants, the adjusted HRs (95% CIs) for incidence of positive CACS comparing sedentary time 7–8 and ≥ 9 h/day to the sedentary time of < 7 h/day were 1.55 (1.06–2.26) and 1.47 (1.06–2.04), respectively. In contrast, in obese participants (BMI ≥ 25 kg/m^2^), there was no significant association between sedentary time and incidence of positive CACS. The *p* value for interaction of obesity and sedentary time on incident positive CACS was 0.380 (Table 3).

**Table 3:** Development of positive coronary artery calcium score (CACS) by sedentary time in obese and non-obese participants.

Sedentary time	Person-years	Number of incident cases	Incidence rate (per 1000 person-years)	Multivariable-adjusted HR^a^ (95% CI)
**BMI ≥ 25 kg/m**^**2**^** (n = 6129)**				
< 7 h/day	5700.8	77	13.51	1.00 (reference)
7–8 h/day	5060.1	65	12.85	1.02 (0.72–1.45)
≥ 9 h/day	13351.2	156	11.68	1.12 (0.83–1.50)
*p* for trend				0.426
**BMI < 25 kg/m**^**2**^** (n = 8820)**				
< 7 h/day	8559.8	62	7.24	1.00 (reference)
7–8 h/day	7585.6	62	8.17	1.55 (1.06–2.26)
≥ 9 h/day	19854.6	147	7.4	1.47 (1.06–2.04)
*p* for trend				0.036
*p* for interaction				0.380

*BMI* body mass index, *CI* confidence interval, *HR* hazard ratio.

^a^Adjusted for age, alcohol intake, smoking status, regular exercise, BMI, systolic blood pressure, glucose, LDL, triglycerides, medication for dyslipideima, and hsCRP level.

## Discussion

We investigated the sedentary time per day in a healthy male group and followed up the calculated CACS, and we found that there was a statistically significant association between prolonged sedentary time and CACS.

Agatston score is most commonly used coronary artery calcium scoring method. Agatston score which is weighted higher for denser coronary artery calcium assumes that both the area and density of calcified plaques are positively related to CVD events^15^. However, recent studies show that higher coronary artery calcium density is associated with lower CVD risk for a given coronary artery calcium volume^15,16^. Of course, because of the high correlation between the Agatston score and other CACS, there are only minor differences in CVD risk prediction with other CACS algorithms. But, Agatston score alone is not optimal measures to use in CVD risk prediction since coronary artery calcium density was inversely associated with incident CVD events^16^.

Several previous studies have investigated the relationship between sedentary time and cardiovascular disease or CACS. Pandey et al.^17^ confirmed that the risk of cardiovascular disease increases when the sedentary time is 10 h or more per day. According to a study by Kulinski et al.^18^, after multivariable adjustment, coronary artery calcium prevalence (CACS > 10) increased by 12% every additional hour in sedentary time. On the other hand, according to a study by Hamer et al.^14^, there was no significant association between sedentary time and CACS. A Swedish study^19^ has also demonstrated that physical activity was effective in reducing significant coronary artery calcium (CACS ≥ 100) in patients with metabolic syndrome, and physical activity and sedentary time were associated with coronary artery calcium and ankle brachial index^20^.

In several studies, occupational physical activity (OPA) and leisure time physical activity (LTPA) differed in their effects on cardiovascular disease. In a prospective cohort study in Belgium^21^, moderate to high LTPA had a significant protective effect against coronary artery disease in low OPA workers but not in high OPA workers. In an Italian study^22^ of adult men aged 25–64 years, the CVD risk increased as the labor intensity increased. Furthermore, sports physical activity had a protective effect for sedentary workers but had no or adverse effects on moderate and strenuous OPA workers. A Danish study^23^ found that persistent LTPA reduced both the coronary heart disease and all-cause mortality. People also spend more time sitting down using electronic devices such as computers and televisions^24^. The increase in television viewing time was small, but it significantly increased mortality from coronary artery disease and total CVD^25^.

In previous studies, the conclusion of the association between prolonged sedentary time or physical activity and CACS is controversial. The reasons are as follows. First, the results may differ depending on whether the sedentary time or physical activity is for rest, leisure, or work. Second, depending on whether the definition of negative CACS is 0 or less than 1, or another criterion, the conclusion may be different. Third, since most of previous studies were cross-sectional studies, the evidence of temporal causality was relatively weak. Finally, because CACS measurement alone cannot exactly identify more vulnerable lesions, further studies are needed to determine whether physical activity can promote plaque stability.

Inflammation caused by apolipoprotein in the arterial wall is critical for the development of atherosclerosis and vascular calcification^6^. Several mediators are associated with inflammation and vascular calcification. Adipokines, such as adiponectin and leptin, and inflammatory cytokines, such as interleukin-6 (IL-6), tumor necrosis factor-alpha (TNF-α), contribute to the regulation of insulin resistance and metabolism of glucose and fat, thus affecting atherosclerosis^26^. They also affect vascular homeostasis and endothelial function. Sedentary behavior decreases the frequency of muscle contraction and reduces shear stress^27^. This causes impairment in glucose metabolism, inhibits lipoprotein lipase, and decreases the bioavailability of nitric oxide. It can also contribute to the development of metabolic diseases by affecting body composition, weight, and dyslipidemia. In addition, sedentary behavior decreases metabolic function by decreasing insulin sensitivity and increasing Insulin-like growth factor 1 (IGF-1) and fasting glucose levels^28^. Another fact to note here is the association between sedentary behavior and chronic inflammation. When sedentary time increases and physical activity decreases, adipokines such as leptin and inflammatory cytokines such as IL-6 and TNF-α increase, leading to chronic low-grade inflammation^28^. As mentioned above, chronic inflammation is critical for the development of atherosclerosis and vascular calcification in the arterial wall.

Obesity is strongly associated with the incidence of CVD and mortality^29^. Meanwhile, sedentary behavior not only increases CACS by contributing to obesity, but also induces coronary artery calcification by decreasing metabolic function and increasing chronic inflammation independently of body fat. This could be known from our results of statistical analysis of the interaction between obesity and sedentary time (*p* value for interaction = 0.380) in addition to the results of previous studies. In our subgroup analysis, the association between prolonged sedentary time and incidence of positive CACS was more pronounced in non-obese participants (BMI < 25 kg/m^2^). In contrast, in obese participants (BMI ≥ 25 kg/m^2^), there was no significant association between sedentary time and incidence of positive CACS. This supports that prolonged sedentary time has a significant association with the incidence of positive CACS independently of obesity.

Our study had several limitations. First, in the questionnaire on sedentary time, there was no distinction between occupational sedentary time and the time spent sitting in leisure time. Previous studies^21,22^ have also shown that there is a difference in the effect of OPA and LTPA on CVDs. However, we supplemented the limitations by including regular exercise in the study and adjusting it in the analysis. Second, participants could answer different categories of sedentary time for each follow-up. However, in our study, nearly half of the participants did not change their answers in terms of categorized sedentary time during follow-up, and two-thirds had changed their answer by less than 1 unit for categorized sedentary time during the entire follow-up period. Third, because the number of female incident cases was too small, it was excluded from the final analysis. It may serve as a limitation to the generalizability. Supplementary tables of the excluded women’s characteristics and the number of incident cases were presented. Finally, because participants self-reported the questionnaire about sedentary time, information bias can occur in the process of subjectively answering the average sedentary time. Nevertheless, this misclassification is likely to be non-differential^30^ and therefore attenuates the association because there is no gain to answering more or less sedentary time.

Our study had several strengths. First, while most previous studies on the relationship between sedentary time and CACS are cross-sectional, our study is a cohort study with a high level of evidence for temporal causality. Second, our study was conducted with a large population, which allowed us to select participants with negative CACS at baseline. Third, our study cohort consisted of relatively young, healthy men who were less affected by underlying diseases. Finally, through subgroup analysis according to obesity, sedentary time was found to have a significant relationship with CACS in metabolically healthy people.

In conclusion, prolonged sedentary time was significantly associated with incidence of positive CACS in the study. CACS is also an effective screening tool for predicting future cardiovascular events in asymptomatic patients. Therefore, CACS can be an effective method for predicting coronary artery diseases in people with prolonged sedentary time, especially in metabolically healthy people.

## Supplementary Information

Supplementary Tables.

## Data Availability

The data are not available to be shared publicly because we do not have a permission from the IRB to distribute the data. However, analytical methods are available from corresponding author on reasonable request.
