# Supplementary material for: The association between prolonged sedentary time and coronary artery calcification in young healthy men in Korea: a cohort study
Source: Sci Rep. 2022 Feb 17;12:2724. doi: 10.1038/s41598-022-06739-x (PMC8854407; doi:10.1038/s41598-022-06739-x)
Supplement: Supplementary file 1 — Supplementary Tables. [file 41598_2022_6739_MOESM1_ESM.docx]

**Supplementary table 1**. Baseline characteristics of female participants by reported sedentary time. [BMI = body mass index; BP = blood pressure; LDL-C = low-density lipoprotein cholesterol; HDL-C = high-density lipoprotein cholesterol; hsCRP = high-sensitivity C-reactive protein]

| Characteristics | Overall | Sedentary time | | | *p* for trend |
| --- | --- | --- | --- | --- | --- |
|  |  | <7 h/day | 7–8 h/day | ≥9 h/day |  |
| Number | 2,376 | 904 | 451 | 1,021 |  |
| Age (years)^a^ | 37.1 (6.7) | 39.7 (6.9) | 36.7 (6.0) | 34.9 (5.9) | <0.001 |
| Current smoker (%) | 1.94 | 1.66 | 1.55 | 2.35 | 0.385 |
| Alcohol intake (%)^b^ | 15.86 | 12.61 | 16.4 | 18.51 | 0.004 |
| Regular exercise (%)^c^ | 10.35 | 15.71 | 7.32 | 6.95 | <0.001 |
| Obesity (%) | 14.02 | 15.27 | 14.63 | 12.63 | 0.099 |
| BMI (kg/m^2^) | 21.9 (3.1) | 22.1 (3.1) | 21.9 (3.3) | 21.7 (3.0) | 0.004 |
| Systolic BP (mmHg)^a^ | 100.5 (10.3) | 101.8 (11.3) | 100.6 (10.8) | 99.3 (9.0) | <0.001 |
| Diastolic BP (mmHg)^a^ | 64.7 (8.3) | 65.1 (8.8) | 64.7 (8.8) | 64.2 (7.5) | 0.015 |
| glucose (mg/dL)^d^ | 90.0  (86-95) | 91.0  (86-95) | 91.0  (86-95) | 90.0  (85-94) | 0.001 |
| Total cholesterol (mg/dL) ^a^ | 188.2 (32.7) | 190.1 (34.1) | 186.8 (32.3) | 187.2 (31.4) | 0.052 |
| LDL-C (mg/dL) ^a^ | 112.8 (30.2) | 114.8 (31.6) | 112.1 (30.4) | 111.4 (28.7) | 0.015 |
| HDL-C (mg/dL) ^a^ | 65.1 (14.8) | 64.9 (15.4) | 65.2 (14.6) | 65.1 (14.4) | 0.779 |
| Triglycerides (mg/dL) ^d^ | 71.0  (55-96) | 72.0  (55-97) | 72.0  (54-96) | 69.0  (54-94) | 0.050 |
| Medication for dyslipidemia (%) | 0.97 | 1.22 | 0.89 | 0.78 | 0.338 |
| hsCRP (mg/L)^d^ | 0.03  (0.02-0.07) | 0.03  (0.02-0.07) | 0.03  (0.02-0.07) | 0.03  (0.02-0.07) | 0.468 |

^a^Data are presented as the means (standard deviation).

^b^Data are presented as percentage (the proportion of participants who intake more than 10g of alcohol a day).

^c^Data are presented as percentage (the proportion of participants who answered that they had vigorous physical activity for more than 3 days per week).

^d^Data are presented as the median (interquartile range), or percentage.

**Supplementary table 2**. Development of positive coronary artery calcium score (CACS) by sedentary time in female participants. [CI = confidence interval; HR = hazard ratio]

| Sedentary time | Person-years | Number of incident cases | Incidence rate (per 1,000 person-years) | Age-adjusted HR | Multivariable-adjusted HR^a^ (95% CI) |
| --- | --- | --- | --- | --- | --- |
| <7 h/day | 3072.3 | 0 | 0 | 1.00 (reference) | 1.00 (reference) |
| 7–8 h/day | 1586.1 | 2 | 1.3 | incalculable | incalculable |
| ≥9 h/day | 3721.1 | 5 | 1.3 | incalculable | incalculable |
| *p* for trend |  |  |  | 0.05 | 0.04 |

^a^Adjusted for age, alcohol intake, smoking status, regular exercise, BMI, systolic blood pressure, glucose, LDL, triglycerides, medication for dyslipidemia, and hsCRP level
